# Annexin A2 in Fibrinolysis, Inflammation and Fibrosis

**DOI:** 10.3390/ijms22136836

**Published:** 2021-06-25

**Authors:** Hana I. Lim, Katherine A. Hajjar

**Affiliations:** 1 Division of Hematology and Oncology, Department of Medicine, Weill Cornell Medicine, New York, NY 10065, USA; hil9005@nyp.org; 2 Division of Hematology and Oncology, Department of Pediatrics, Weill Cornell Medicine, New York, NY 10065, USA; 3 Department of Cell and Developmental Biology, Weill Cornell Medicine, New York, NY 10065, USA

**Keywords:** annexin A2, fibrinolysis, inflammation, fibrosis, thrombosis, autoimmune disease, angiogenesis, tissue repair

## Abstract

As a cell surface tissue plasminogen activator (tPA)-plasminogen receptor, the annexin A2 (A2) complex facilitates plasmin generation on the endothelial cell surface, and is an established regulator of hemostasis. Whereas A2 is overexpressed in hemorrhagic disease such as acute promyelocytic leukemia, its underexpression or impairment may result in thrombosis, as in antiphospholipid syndrome, venous thromboembolism, or atherosclerosis. Within immune response cells, A2 orchestrates membrane repair, vesicle fusion, and cytoskeletal organization, thus playing a critical role in inflammatory response and tissue injury. Dysregulation of A2 is evident in multiple human disorders, and may contribute to the pathogenesis of various inflammatory disorders. The fibrinolytic system, moreover, is central to wound healing through its ability to remodel the provisional matrix and promote angiogenesis. A2 dysfunction may also promote tissue fibrogenesis and end-organ fibrosis.

## 1. Introduction

As essential components of hemostasis, the fibrinolytic and coagulation systems maintain a critical balance between clot formation and the dissolution of thrombi. The coagulation cascade, which is typically activated by tissue or vascular injury, generates thrombin, which converts soluble fibrinogen into insoluble fibrin during clot formation. Thereafter, the controlled activation of plasminogen generates plasmin, the main protease that dissolves the insoluble fibrin mesh. The homeostatic balance between coagulation and fibrinolysis requires the regulated activation and deactivation of several serine proteases, thereby enabling hemostasis, vessel healing, and the restoration of vascular patency.

The three pillars of fibrinolysis, plasminogen, tissue plasminogen activator (tPA), and urokinase (uPA), were identified in the last century, and studied extensively in genetic mouse models and in human subjects [1]. Plasminogen, which is the inactive, zymogen form of plasmin, circulates in the blood in a fluid state and can be activated by tPA on fibrin surfaces. In addition to fibrin-based fibrinolysis, there is a more recent understanding that a portion of fibrinolysis occurs through fibrinolytic receptors on cell surfaces. An example is the urokinase plasminogen activator surface receptor (uPAR), which appears to be expressed on migrating endothelial cells [2], as well as many malignant tumors [3]. Localization of uPA by uPAR allows plasmin to be generated within the pericellular milieu, which likely contributes to intravascular fibrinolysis in some settings. uPAR also plays a major role in degradation of the extracellular matrix by other migrating cells, including macrophages, smooth muscle cells, myeloid progenitors, and tumor cells [4,5,6].

Our group and others identified another important cell surface fibrinolytic receptor, the annexin A2 (A2) complex [7]. A2 is a calcium-sensing, phospholipid-binding protein that forms a heterotetramer with its binding partner S100A10, also known as p11. On the endothelial cell surface, the heterotetramer A2 complex binds plasminogen and tPA, facilitating the activation of the main fibrinolytic protease, plasmin [8]. Similar to uPAR-bound, uPA-generated plasmin, cell surface A2-generated plasmin appears to not only clear fibrin, but also promote proteolysis of the extracellular matrix. For example, endothelial cells migrating away from existing blood vessels appear to use this system to create new vasculature in the process of angiogenesis [9].

In both its heterotetrameric and monomeric forms, A2 is produced by a wide array of other cell types, including trophoblast, epithelial, dendritic, and tumor cells, as well as macrophages and monocytes. In addition to maintaining cell surface proteolytic activity, A2 also fulfills a range of intracellular functions, including membrane repair, exocytosis, endocytosis [10], and maintenance of adherens-like intercellular junctions [11]. Its numerous functions contribute to fibrinolysis, regulation of inflammation and immune system activation, and tissue injury and repair; as a result, A2 dysfunction has been implicated in multiple human diseases.

## 2. A2 in Hemostasis and Vascular Homeostasis

The annexins are a family of Ca^2+^-regulated, phospholipid- and membrane-binding proteins that have unique Ca^2+^ binding sites located within the highly alpha-helical “annexin” repeats within the core domain of the molecule [12]. This architecture allows annexins to interact with phospholipids and bridge disparate membranes, or connect membranes to other intracellular structures [13]. Some annexins form a complex with members of the S100 family of Ca^2+^ binding proteins, as exemplified by the interaction of S100A10 (protein p11) with A2. On the cell membrane, A2 associates via its N-terminal tail with the C-terminal region of p11 to form a heterotetrameric complex (A2●p11)_2_. Influx of Ca^2+^ into the intracellular space of the endothelial cell mobilizes (A2●p11)_2_ to the inner surface of the plasma membrane [14]. Many functions of A2 are regulated by phosphorylation [15], including phosphorylation of Tyr23 residue on A2 by the src family kinase, pp60src, which is required for A2 translocation to the cell surface in these cells [16].

The fibrinolytic receptor function of A2 was discovered after identification of a ~36-kDa, tPA- and plasminogen-binding protein on the endothelial cell surface [7,17]. The (A2●p11)_2_ complex was found to bind plasminogen specifically at the cleaved C-terminus of A2 [18] and, in a purified protein system, to accelerate the catalytic efficiency of tPA-dependent plasmin generation ~60-fold [19]. In later studies, the tPA-binding domain was identified within the N-terminal tail of A2 [20]. This profibrinolytic assembly on the endothelial cell surface was then theorized to play an important role in maintaining vascular and hemostatic homeostasis in vivo [21,22].

Development of a murine model of global A2 deficiency (*Anxa2^−^*^/*−*^) revealed several key physiologic roles for A2 [23]. Similar to mice with classical deficiency of plasminogen or both uPA and tPA, A2-deficient mice exhibited a two- to three-fold increase in fibrin accumulation in organs such as the lungs, heart, and kidneys compared to wild-type tissues. Moreover, *Anxa2^−^*^/*−*^ mice showed impaired clearance of injury-induced arterial thrombi, though to a lesser degree than that observed in complete plasminogen or plasminogen activator deficiency. In addition to surface fibrinolysis, in vitro models demonstrated that A2 also contributes to secretion of prohemostatic factors such as von Willebrand factor (vWF) by vascular endothelial cells. Regulation of the Munc13-4/(A2●p11)_2_ complex through dephosphorylation of A2 appears to mediate Ca^2+^-evoked exocytosis of Weibel–Palade bodies [24,25].

Given these implications for hemostasis, it was predicted that the overexpression or gain-of-function of surface A2 would lead to hemorrhage, and that the underexpression or loss-of-function of surface A2 would result in thrombosis in humans. The first “annexinopathy” [26] was described in acute promyelocytic leukemia (APL). Compared to blast cells from other forms of leukemia, APL leukemic cells exhibit strikingly increased intracellular and cell surface A2 expression, resulting in increased plasmin generation [27,28,29]. Excessive A2-related plasmin generation leads to the rapid consumption of fibrinolytic inhibitors and unregulated fibrinolysis. This, together with disseminated intravascular coagulopathy initiated by the release of procoagulants from the leukemic cells, leads to the hemorrhagic diathesis of APL.

Furthermore, autoantibodies against A2 were found in antiphospholipid syndrome (APS) with thrombosis. These autoantibodies block endothelial surface tPA-dependent plasmin generation [30]. Similarly, high-titer anti-A2 antibodies were found in 12.5% of a Mexican Mestizo population with cerebral venous thrombosis [31], and anti-A2 IgG antibodies were associated with a high risk of thrombosis and/or pregnancy morbidity in Chinese patients with APS and systemic lupus erythematosus (SLE) [32]. Most recently, our group identified for the first time that a cohort of patients with a positive personal and family history of thrombosis, but without a known inherited thrombophilia, had significantly low expression of A2 and reduced cell surface plasmin generation, demonstrating that A2 deficiency may represent a novel risk factor for inherited thrombophilia [33].

**Figure 1:**
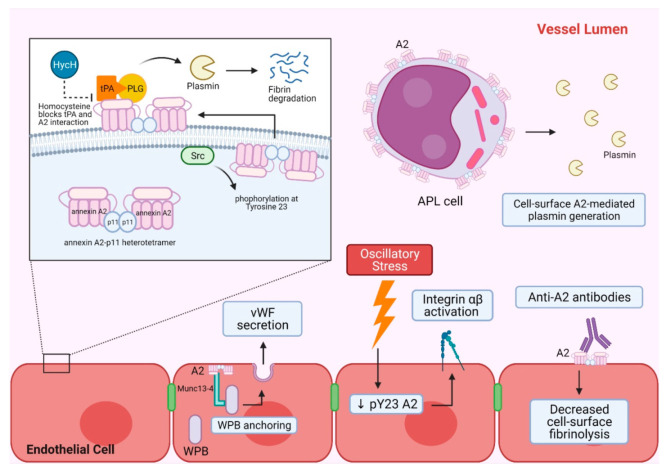
A summary of A2-mediated hemostasis and vascular homeostasis. A2 heterotetramer and its main cell surface fibrinolytic function are shown in the top left panel. A2 forms a heterotetramer with p11 intracellularly and associates with the inner leaflet of the phospholipid bilayer of the plasma membrane. When A2 is phosphorylated at tyrosine 23 by Src kinase, the heterotetramer is translocated to the extracellular membrane and functions as a tPA and PLG receptor for efficient plasmin generation leading to fibrin degradation. Homocysteine can block the interaction between tPA and A2, and decrease A2-mediated plasmin generation. The top right shows APL cells with increased cell surface A2, which can lead to increased plasmin generation and unopposed fibrinolysis. The bottom section shows other roles of A2 in endothelial cells that contribute to hemostasis. A2 facilitates WPB anchoring and subsequent exocytosis of its content by interacting with Munc13-4. WPB exocytosis results in vWF secretion. Oscillatory stress on endothelial cells can downregulate phosphorylated A2, which in turn activates integrin αβ. Autoantibodies against A2 have been shown to bind to extracellular surface A2 and decrease cell surface fibrinolysis. A2 = annexin A2, APL = acute promyelocytic leukemia, HycH = Homocysteine, p11 = S100A10 protein, pY23 A2 = phosphorylated annexin A2, PLG = plasminogen, Src = Src kinase, tPA = tissue plasminogen activator, vWF = vonWillebrand factor, WPB = Weibel–Palade body.

In addition to venous thrombosis, A2 appears to play a role in the development of atherosclerosis and atherothrombosis. Incubation of endothelial cells with homocysteine, a known macrovascular atherothrombotic agent, resulted in decreased A2-dependent cell surface plasmin generation [34]. Homocysteine reacts with Cys8 within the N-terminal domain of A2 through a disulfide linkage, thereby blocking tPA’s interaction with A2 [35]. Mice subjected to a high-methionine, low-folate diet had an increased plasma level of homocysteine, and exhibited microvascular fibrin accumulation similar to that of *Anxa2^−^*^/*−*^ mice [36]. In addition, a recent study showed that mechanical stress on endothelial cells may lead to atherosclerosis through an interaction between intracellular A2 and integrin α5. Disturbed flood flow with oscillatory stress on endothelial cells accelerated the dephosphorylation of A2, which facilitated the binding of integrin α5 to the C-terminal domain of A2, thereby activating integrin α5β1, and leading to the progression of atherosclerosis [37,38]. Mechanisms of A2-mediated hemostasis and vascular homeostasis are summarized in Figure 1.

The use of recombinant A2 (rA2) as a potential therapeutic adjunct to conventional thrombolytic therapies has been explored by several groups in preclinical studies. In a rat carotid artery thrombosis model, pretreatment of animals with an intravenous rA2 monomer enhanced the patency of the carotid arteries, resulting in reduced thrombus formation [39]. In a rat embolic stroke model, infusion of rA2 alone increased relative blood flow and reduced the size of the final infarct [40]; coadministration of rA2 also enhanced the thrombolytic activity of tPA when used at lower doses [41]. The combination of low-dose tPA with rA2 also improved long-term neurologic function one month after the induction of a stroke [42] and reduced early blood–brain barrier disruption in the rat embolic stroke model [43] without significant hemorrhagic transformation. These data suggest that rA2 may be a viable antithrombotic therapy for humans in the future.

## 3. A2 in Inflammation and Autoimmune Disorders

Inflammation is a complex, protective host reaction to harmful stimuli, such as infection or injury, resulting in the activation of local and systemic processes to eliminate or compartmentalize the inciting agent. A2 fulfills a number of roles at different stages of inflammation and may contribute to a variety of human disease states related to its acute and chronic forms [44]. 

When a pathogen or an insult is introduced, the earliest inflammatory response is a loss of vascular integrity and recruitment of immune cells into the affected tissue. Normally, vascular permeability is tightly regulated by ensuring endothelial–endothelial cell junction stability through homotypic interactions of several junctional proteins, including vascular endothelial cadherin (VEC), a major component of the adherens type of junction [45]. Src kinase-mediated tyrosine phosphorylation of VEC induces the opening of adherens junctions, and (A2●p11)_2_ interacts directly with VEC via actin filaments to stabilize the adherens junction [46]. Vascular endothelial growth factor (VEGF) treatment disrupts this interaction, thereby increasing vascular permeability. Within the pulmonary microvasculature, which is highly dependent upon adherens-type junctions, alveolar hypoxia induced pulmonary edema and neutrophilic infiltration in *Anxa2^−^*^/*−*^, but not *Anxa2^+^*^/*+*^, mice [11]. Further investigation revealed that the interaction between A2, VEC, and Src homology phosphatase 2 enables the dephosphorylation of VEC, thus closing adherens junctions; in the absence of A2, cell–cell junctions were unstable and vascular leak ensued.

Loss of vascular integrity may be a key mechanism that explains the severe pulmonary sequelae seen in the novel coronavirus infection (COVID-19) due to the SARS-CoV-2 virus. Sera from patients with SARS-CoV, as distinct from SARS-CoV-2, contain antibodies that react with several antigens on lung epithelial cells including A2, which displayed the highest immunoreactivity score. Antibodies against the SARS spike domain (S2) bound to A2 and anti-A2 IgG from other sources bound to S2, suggesting shared immunologic epitopes on the two proteins. The binding of anti-S2 to cultured lung epithelial cells was increased by pretreatment with SARS-associated cytokines, which also increased A2 expression [47]. In addition, a recent preliminary study revealed higher average levels of anti-A2 antibodies in hospitalized COVID-19 patients who died, when compared with non-critical hospitalized COVID-19 patients and critically ill non-COVID-19 patients [48].

Once vascular integrity is lost, inflammatory leukocytes are recruited to the site of insult. The chemotaxis of monocytes may partly depend on A2, and anti-A2 antibodies, or downregulation of A2, dampen plasmin-mediated chemotaxis of monocytes [49]. Monocytes and monocyte-derived macrophages express high levels of A2 on the cell surface, unlike granulocytes, and the surface A2 facilitates cytokine-directed monocyte/macrophage migration through the extracellular matrix [50], further mobilizing monocytes to the site of active inflammation. A2, through an interaction with CD44, may also promote neutrophil chemotaxis in the presence of complement-activated serum and vitamin D-binding protein; anti-A2 appears to block this effect on human neutrophil chemotaxis [51].

**Figure 2:**
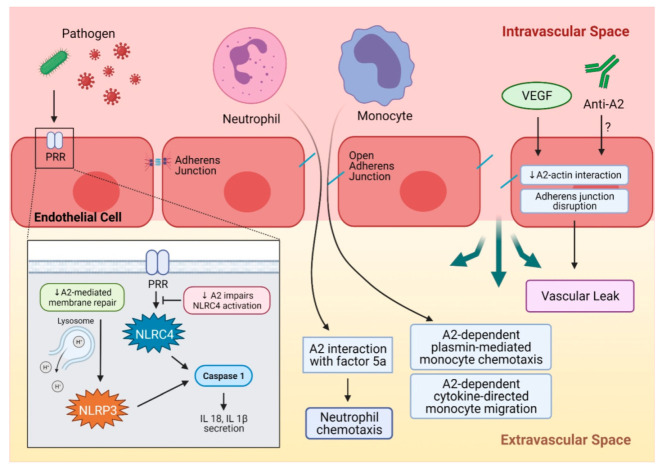
A summary of A2 in inflammation. Pathogens such as bacteria or viruses are recognized by PRR and activate NLRC4, which then activates caspase 1 and leads to IL-18 and IL-1β secretion. A decrease in A2 impairs NLRC4 activation by PRR. Another inflammasome, NLRP3, can be activated by endolysosomal damage and the leakage of its contents, and results in caspase activation and pro-inflammatory cytokine secretion. A2 plays a critical role in endolysosomal membrane repair, and downregulation of A2 can impair endolysosomal membrane repair and activate the NLRP3 inflammasome. Inflammatory cells like neutrophils and monocytes can be recruited to a site of injury or insult by A2-mediated mechanisms. Neutrophil chemotaxis to the injury site occurs through A2 interaction with activated complement factor 5a. Monocytes can be recruited by A2-dependent, plasmin-mediated chemotaxis, and an A2-dependent cytokine-directed mechanism facilitates monocyte migration. A2 maintains integrity of adherence junctions through interaction with actin, and this can be interrupted by VEGF, leading to adherens junction disruption and vascular leak. A similar mechanism can be proposed for anti-A2 autoantibodies that can disrupt A2-actin interaction. A2 = annexin A2, NLRC4 = NLR family CARD-containing protein 4 inflammasome, IL-18 = interleukin-18, IL-1β = interleukin-1β, NLRP3 = NLR family pyrin domain containing 3 inflammasome, PRR = pattern recognition receptor, VEGF = vascular endothelial growth factor.

Inflammasomes, which are multicomponent cytosolic protein complexes that regulate responses to the activation of pattern-recognition receptors (PRR) within inflammatory cells such as macrophages, monocytes, dendritic cells, and neutrophils, appear to have a dynamic spectrum of interactions with A2. Activation of PRRs triggers signaling cascades and downstream activation of caspase-1 that cleaves proinflammatory cytokines, thus enabling their secretion [52]. In addition to external pathogens, caspase-1 responds to other cellular stresses, such as leakage of lysosomal contents, mitochondrial damage, and production of reactive oxygen species (ROS). A2-null macrophages infected with *Anaplasma phagocytophilum* showed evidence of impaired NLRC4 inflammasome activation, resulting in defective caspase-1 activation, and reduced interleukin-1β (IL-1β) and IL-18 secretion [53]. On the other hand, the involvement of A2 in endolysosomal membrane repair appears to prevent NLRP3 inflammasome activation. In humans, wear debris particles from joint replacement devices are phagocytosed by dendritic cells and may damage the endolysosomal membrane. Damage to these membranes releases cathepsins and H^+^ ions into the cytosolic space, leading to activation of the NLRP3 inflammasome. In the cytosol of dendritic cells exposed to wear debris particles, profound downregulation of A2, and translocation of both A2 and p11 to the endosomes was observed, preventing the resealing of the damaged membranes by A2 complex and local microvesicles, thereby increasing inflammasome activation [54]. In the cecal ligation model of sepsis, on the other hand, *Anxa2^−^*^/*−*^ mice manifested a more severe sepsis phenotype with elevated IL-17 and increased ROS [55]. Stabilization of the inflammasome may reflect a direct ROS neutralizing effect of A2, with reduced inflammasome-mediated proinflammatory cytokine production. Overall, it appears that A2 may play a spectrum of roles in varying inflammatory settings that trigger different pathways within the multifunctional inflammasome system (Figure 2).

The complement system is a protease cascade that generates potent proinflammatory mediators, opsonins, and membrane-penetrating pores within the innate immune system [56]. One of three complement-activating mechanisms, the alternative pathway (AP), can be initiated in the fluid phase and also on cell surfaces, and is amplified by circulating factor H. Proteomic analysis of ischemic kidney tissue in mice revealed that A2 can bind to factor H, and administration of recombinant A2 increased complement activation in oxygen-deprived glomeruli [57]. A2 may also trigger activation of the classical complement pathway by binding to C1q on apoptotic cells [58]. These findings may explain how local factors, such as high A2 in renal glomeruli, contribute to human complement disorders such as atypical hemolytic uremic syndrome.

In human autoimmune disorders, dysregulation of adaptive immune responses by autoantibodies is a common pathophysiologic mechanism. The mechanistic role of many of these autoantibodies is currently being studied. In antiphospholipid syndrome, for example, the anti-β_2_ glycoprotein antibody has been shown to bind to A2 with high affinity and may activate endothelial cells [59,60,61]. Direct anti-A2 antibody formation also occurs in patients with antiphospholipid syndrome, systemic lupus erythematosus, and other autoimmune disorders [30,32,62], and may contribute to the thrombotic outcomes in these diseases [63]. In addition, anti-dsDNA antibodies, considered to be a predictive marker of lupus nephritis, were observed to interact with A2 on mesangial cells [64]. A2 was found to colocalize with immune deposits in human and murine lupus nephritis biopsies, further suggesting a pathogenic interaction between anti-dsDNA and A2, which may subsequently induce IL-6 secretion. In summary, A2 impacts many stages of inflammation, leading to immune dysregulation and end-organ injury in autoimmune disorders.

## 4. A2 in Tissue Repair and Fibrosis

A2 may play a role in the resolution of tissue injury caused by chronic tissue inflammation, oxidative damage, and hypoxia through its roles in tissue repair processes, including cell migration and angiogenesis. For example, chronic muscle inflammation and degeneration are hallmarks of symptomatic muscular dystrophies, and mutations in a crucial protein called dysferlin lead to limb-girdle muscular dystrophy type 2B and Miyoshi myopathy. Dysferlin interacts with A2 in the sarcolemmal repair process by facilitating intracellular F-actin accretion at the site of injury. In fact, skeletal muscle from A2-null mice demonstrated poor myofiber repair [65]. In a follow-up study, A2 responded to the injury-triggered increase in cytosolic Ca^2+^ by accumulating beneath the injured sarcolemma and promoting fusion of dysferlin-rich vesicles to repair damaged membranes [66]. Tyrosine phosphorylation of A2 in baby hamster kidney cells, moreover, regulates actin rearrangement and cell morphology changes [67], further suggesting that the ability of A2 to modulate the cytoskeletal structure may regulate cellular attachment. A2 also facilitates endocytosis of β1-integrin in intestinal epithelial cells, and a loss of A2 inhibited cell migration and enhanced cell-matrix adhesion [68]. In this study, A2 also appeared to facilitate migration of a cohesive epithelial sheet and subsequent mucosal wound closure by promoting cell detachment.

Reactive oxygen species (ROS) are a class of compounds, including hydrogen peroxide, superoxide, hydroxyl radical, and singlet oxygen, that are capable of damaging cellular macromolecules, including proteins, lipids, and DNA. ROS also participate in receptor signaling, cell proliferation, apoptosis, and inflammasome activation. A2 has been suggested to function as a redox protein by reversible oxidation of its Cys8 residue [69]. When exposed to H_2_O_2_, phosphorylated A2 (pTyr23A2) located in the promyelocytic leukemia (PML) bodies of the nucleus became dephosphorylated, whereas F-actin-associated A2 at the cell cortex became phosphorylated in rat pheochromocytoma cells. These responses were transient, and the pTyr23A2 at the cell cortex was subsequently incorporated into vesicles and released into the extracellular space [70], hinting at the possibility that ROS-induced exocytosis of cellular components can be mediated by A2. H_2_O_2_ treatment of non-cancerous breast epithelial cells caused IL-1α and A2 to colocalize within lamellipodia, especially at the outer edges of the cell clusters, called “islands”, suggesting the participation of the two proteins in communication with neighboring cells [71].

Dysregulation of the injury-repair response can result in the persistence of fibroblasts and the deposition of collagen, leading to fibrotic scar formation within organs and tissues. A2 was detected in the conditioned media of human lung fibroblast cultures, and incubation of these cells with human plasma or purified factor Xa (FXa) elicited a fibrogenic response. This was attenuated by FXa inhibition with apixaban or anti-A2 antibodies [72]. Extravasation of FXa into the parenchyma following a vascular leak in lung injury, and the subsequent interaction between FXa and A2, appeared to activate fibroblasts and potentiate fibrosis of the lung. This may be a significant mechanism of fibrosis in idiopathic pulmonary fibrosis.

A2 may play a role in fibrosis and cardiac remodeling in the heart, as well. In the hearts of hypertensive guinea pigs, A2 accumulated in the interstitium of the hypertrophied left ventricles [73]. Similar findings of increased A2 mRNA and interstitial protein localization also occurred in failing human hearts [74]. The ability of A2 to bind extracellular collagen and promote cell-cell adhesion might be an important feature in post-injury remodeling of the myocardial interstitium, and the prevention of perivascular cardiac fibrosis [75].

Hepatic fibrosis is a common response to chronic liver injury due to alcohol or viral hepatitis, and A2 may contribute to the progression of cirrhosis. In earlier gene expression profiling studies of alcoholic liver disease in humans and primates, A2 was overexpressed on the hepatocyte surface, proliferative bile duct cells, and a CD14-positive, CD68-negative subpopulation of macrophages [76]. In an immune liver fibrosis model of rats, hepatocyte plasma membrane-associated A2 increased as fibrosis worsened. Similar findings were seen in human liver with extensive fibrosis due to hepatitis B, indicating the potential role of A2 as a biomarker for liver fibrosis [77]. In the rat CCl_4_-induced liver injury model, A2 and vWF were upregulated in the fibrotic liver. Reduction of A2 by RNA silencing (siRNA) in hepatocellular and liver fibrosis cell lines significantly reduced vWF secretion, suggesting that hepatocyte-driven vWF secretion, in addition to the release of vWF by endothelial cells, may contribute to liver fibrosis [78]. In related work, microvascular occlusion due to thrombotic microangiopathy has been proposed as a molecular mechanism for hepatic fibrosis, with increased synthesis of von Willebrand factor (vWF) by endothelial cells or hepatocytes playing a vital role. A2 may modulate vWF secretion by hepatocyte in liver injury, leading to microvascular thrombi and subsequent fibrosis. Together, these studies suggest that A2 may be involved in hepatic fibrosis through a variety of mechanisms.

Completion of the healing process requires angiogenesis for new tissue growth and perfusion. A2-null mice displayed significantly reduced blood vessel formation in postnatal angiogenesis models, suggesting a link between A2-mediated cell surface plasmin generation and angiogenesis [23]. When further studied in the murine oxygen-induced retinopathy (OIR) model, the effect of retinal ischemia and the subsequent angiogenic response was attenuated in *Anxa2^−^*^/*−*^ retina. A hypoxia inducible factor was found to interact with the A2 promoter region for its activation, leading to increased A2 expression in hypoxia-related injury and stimulating angiogenesis [79]. 

**Figure 3:**
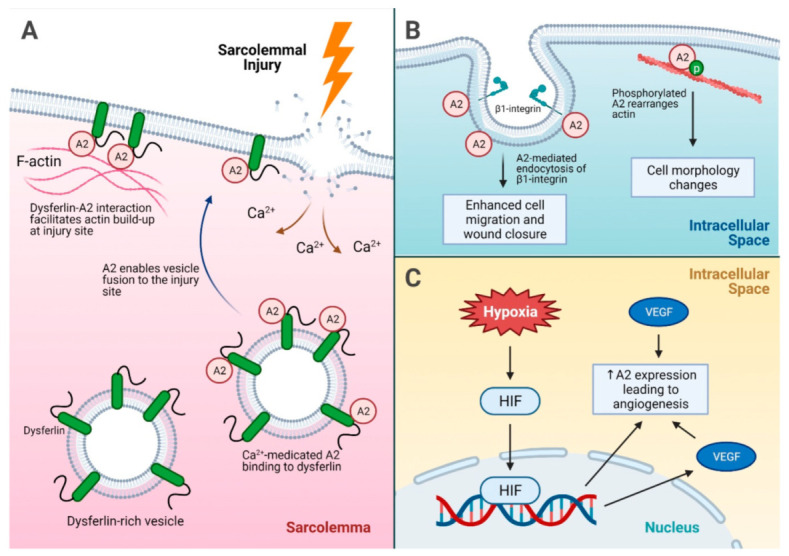
A summary of injury repair mechanisms driven by A2. (**A**) Sarcolemmal injury and the role of A2 in injury site repair. Dysferlin interacts with the A2 intracellular membrane and facilitates actin build up at the injury site. In addition, calcium influx into the sarcolemma after an injury causes A2 binding to dysferlin on dysferlin-rich vesicles, which subsequently fuse to the injury site for membrane repair. (**B**) The mechanisms of cell migration for injury repair facilitated by A2. A2 enhances endocytosis of β1-integrin, which promotes cell detachment and migration for wound closure. In addition, phosphorylated A2 helps rearrange actin, leading to cell morphology changes and modulation of cellular attachment. (**C**) A2-mediated angiogenesis under hypoxic stress. Hypoxia leads to increased A2 expression via direct HIF-induced expression, and HIF can induce VEGF expression, which indirectly leads to increased A2 expression as well. Increased VEGF alone can also lead to overexpression of A2, which promotes angiogenesis. A2 = annexin A2, HIF = hypoxia inducible factor, p = phosphorylation, VEGF = vascular endothelial growth factor.

Other hypoxia- and wound healing-related factors, such as vascular endothelial growth factor (VEGF), have also been shown to stimulate angiogenesis by upregulating A2. Treatment with VEGF increased A2 expression in the mouse model of ischemia-induced retinal neovascularization, and knockdown of A2 by RNA silencing resulted in the suppression of neovascularization in the retina [80]. In addition to the direct A2 induction by VEGF and HIF, indirect upregulation of A2 by HIF-stimulated VEGF was also reported in osteoblastic cells [81]. The ability of A2 to promote angiogenesis is exploited by certain tumor cells, such as highly vascular triple-negative breast cancer tumors in nude mice, in which intravenous injection of an anti-A2 antibody significantly inhibited tumor growth [82,83], and knockdown of A2 in glioma cells reduced angiogenesis in a rodent model of central nervous system glioma [84]. Together, these studies suggest that A2 plays a key role in the physiologic response to tissue injury and wound healing (Figure 3), but can also participate in pathogenic responses such as tissue fibrosis, retinal neovascularization, and tumor angiogenesis [85].

## 5. Conclusions

**Figure 4:**
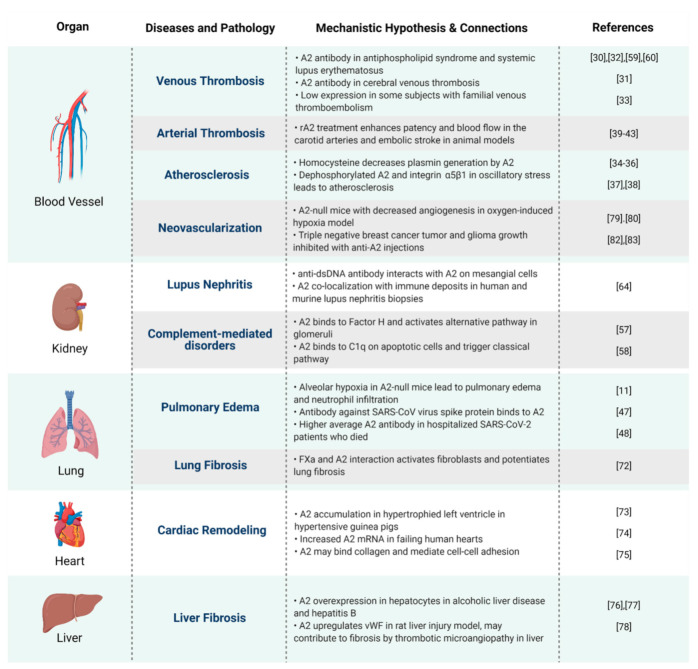
A mechanistic hypothesis and connections between A2 and human diseases. Proposed pathophysiologic mechanisms by which annexin A2 impacts health and disease, organized by a major organ system.

A2 participates not only in cell surface fibrinolysis, but also in various cellular functions that regulate the inflammatory response, injury signaling, and wound healing, thus implicating it in multiple human pathologies (Figure 4). There is abundant evidence that other components of the fibrinolytic system, such as fibrin, plasminogen activator inhibitor-1, and plasmin, engage in tissue damage repair and protection against tissue fibrosis [86,87]. Fibrosis appears to be an outcome of a dysregulated tissue repair response, especially in chronic inflammation [88,89] and, while the complete role of A2 in fibrosis has yet to be elucidated, there is evidence that A2 may participate, either directly or indirectly, in fibrogenesis in various organs. Given this level of complexity, it is reasonable to propose that the fibrinolytic activity of A2 may either promote or protect against pathogenic fibrosis, and its dysfunction or deficiency may contribute to human disease. Clearly, additional studies are needed to investigate this further.

## Data Availability

No new data were created or analyzed in this study. Data sharing is not applicable to this article. Original data can be found in references cited.

## Abbreviations

A2	annexin A2
tPA	tissue plasminogen activator
uPA	urokinase plasminogen activator
APL	acute promyelocytic leukemia
IgG	immunoglobulin G
IL	Interleukin
APS	antiphospholipid syndrome
VEC	vascular endothelial cadherin
VEGF	vascular endothelial growth factor
COVID-19	corona virus disease-19
SARS	severe acute respiratory syndrome
PRR	pattern recognition receptor
ROS	reactive oxygen species
AP	alternative pathway of complement
PML	promyelocytic leukemia
CD	cluster of differentiation
vWF	von Willebrand factor
OIR	oxygen-induced retinopathy
HIF	hypoxia-inducible factor
